# Comparative evaluation of three herbal extracts on growth performance, immune response, and resistance against *Vibrio parahaemolyticus* in *Litopenaeus vannamei*

**DOI:** 10.1186/s12917-025-04588-0

**Published:** 2025-03-13

**Authors:** Amr Fadel, Amal Khafage, Mohamed Abdelsalam, Mohamed M. Abdel-Rahim

**Affiliations:** 1 National Institute of Oceanography and Fisheries (NIOF), Cairo, Egypt; 2 Department of Aquatic Animal Medicine and Management, Faculty of Veterinary Medicine, Cairo University, P.O. 11221, Giza, Egypt

**Keywords:** *Litopenaeus vannamei*, Herbal extracts, Growth performance, Immunity, Histopathology, *Vibrio parahaemolyticus*, AHPND

## Abstract

This study evaluated the effects of dietary supplementation with herbal extracts from *Artemisia herba-alba, Lonicera japonica*, and *Lilium candidum* on growth performance, survival, feed utilization, antioxidant capacity, and immune response in *Litopenaeus vannamei*. The efficacy of these herbal-supplemented diets was assessed in enhancing resistance against *Vibrio parahaemolyticus*-induced Acute Hepatopancreatic Necrosis Disease (*Vp*_AHPND_). A total of 2,400 shrimp post-larvae (initial weight 0.74 ± 0.02 g) were randomly assigned to four triplicate groups. Shrimp were fed isonitrogenous and isocaloric diets: T1 (control, basal diet), T2 (basal diet + 250 mg/kg *A. herba-alba*), T3 (basal diet + 250 mg/kg *L. japonica*), and T4 (basal diet + 250 mg/kg *L. candidum*). Herbal-supplemented groups showed significantly improved (_*P*_ ≤ *0.05*) growth performance, feed utilization, and survival rates compared to the control, with T4 exhibiting the highest values. Significant enhancements of immune assays were observed in total hemocyte count, phagocytosis activity, total protein, glutathione peroxidase, and lysozyme activity in herbal-supplemented groups. Antioxidant indicators (catalase, superoxide dismutase, and phenoloxidase) were boosted while malondialdehyde levels decreased in herbal-treated shrimp. Following *V. parahaemolyticus* challenge, herbal diets effectively reduced cumulative mortality in *L. vannamei*. Histopathological examination revealed milder AHPND-associated alterations in *A. herba-alba* and *L. candidum*-treated groups, contrasting with atrophy, necrosis, and epithelial cell sloughing observed in the positive control. These findings demonstrate the immunostimulatory potential of *A. herba-alba, L. japonica*, and *L. candidum* as dietary supplements to enhance growth performance, immune function, and disease resistance in *L. vannamei* aquaculture, offering a promising strategy for sustainable shrimp farming.

## Introduction

Aquaculture has emerged as one of the most promising food-producing sectors, demonstrating the greatest capacity to meet rising global food demand [1, 2]. Among the cultivated species, *Litopenaeus vannamei* stands out as an omnivorous and vigorous shrimp species, widely distributed and exhibiting robust environmental adaptability and rapid growth [3, 4]. Over the past two decades, *L. vannamei* cultivation has experienced exponential growth worldwide, becoming the most significant commercial aquaculture industry [5–7]. However, the expansion of shrimp culture practices has exacerbated various threats, including environmental degradation, disease emergence, and devastating impacts on the industry [8, 9].

Vibriosis, a serious bacterial disease in shrimp aquaculture, continues to impede the progress of shrimp farming, leading to substantial economic losses [6, 10, 11]. Several pathogenic *Vibrio* species have been identified as causative agents of Vibriosis in shrimp, including *V. harveyi, V. anguillarum, V. splendidus, V. parahaemolyticus,* and *V. alginolyticus* [11, 12]. Of particular concern is Acute Hepatopancreatic Necrosis Disease (AHPND), an emerging bacterial disease caused by *V. parahaemolyticus Vp*_AHPND_. AHPND is considered one of the most dangerous diseases affecting farmed shrimp, with mortality rates reaching 70–100% [13–15]. Infected shrimp with AHPND display symptoms such as lethargy, poor growth, empty stomachs, pale hepatopancreas, reddish carapace, and redness of the swimming and walking legs [15, 16]. In Egypt, Vibriosis outbreaks have been documented, causing mortalities in *L. vannamei* cultured in earthen ponds [17]. Moreover, *V. parahaemolyticus* infections pose additional threats due to their zoonotic potential and increasing concerns regarding multi-resistant bacteria [18].

Traditionally, chemotherapeutics, including chemicals, antibiotics, and disinfectants, have been used to prevent and treat AHPND [13, 19]. However, the overuse of antimicrobials has resulted in resistant bacteria and antibiotic residues in food, posing dangers to both the aquatic environment and human health [20]. In response to these challenges, eco-friendly aquaculture models have been proposed to enhance immune responses, control disease outbreaks, and improve aquaculture production. These models incorporate prebiotics, probiotics, synbiotics, and algal and herbal phytobiotics [1, 21–23].

Herbal extracts have gained attention as potential alternatives to chemotherapeutics due to their safety, ease of use, high effectiveness, and eco-friendly nature for both farmed animals and humans [24]. These extracts offer numerous benefits to aquatic animals, including enhanced immune system function, stimulated growth and maturation, inhibition of pathogenic bacteria, viruses, fungi, and parasites, and reduced stress [25, 26]. The bioactive compounds in herbal extracts can promote physiological metabolism, and protein synthesis, improve growth performance and feed utilization, and even enhance the quality of aquatic products [27]. In shrimp aquaculture, certain herbal plants, such as *myrtle* leaf extract and *Eclipta alba*, have demonstrated protective effects against various pathogenic *Vibrio* species and AHPND-causing *V. parahaemolyticus Vp*_AHPND_, thereby reducing the incidence of AHPND [28–30].

This study evaluates three herbal extracts as potential immunostimulants, growth promoters, and antibacterial agents in shrimp culture: *Artemisia herba-alba*, *Lonicera japonica* Thunb, and *Lilium candidum* L. *A. herba-alba*, whose main active ingredient is Artemisinin (ART), has shown promising pharmacological effects, including antibacterial [31], antiviral, and anti-inflammatory properties, which could be beneficial in addressing shrimp diseases [31]. Artemisinin (ART) possesses antioxidant capacity through the activation of CAT, SOD, and GSH-Px creating a reduction of lipid peroxidation [29]. In addition, ART-supplemented diets enhanced the non-specific immunity markers including the phenol oxidase, lysozyme, and alkaline phosphatase [32]. *Lonicera japonica* Thunb, a species of honeysuckle, has been used in traditional medicine for centuries and has recently gained attention for its potential antimicrobial and immunomodulatory properties [33]. *Lonicera* has been proven their immunostimulant, antimicrobial, antiviral, anti-inflammatory, antioxidant, and hepatoprotective activities associated with major active compounds including flavonoids, glycosides, iridoids, triterpenoids, organic acids, and lignans, volatile oil providing [34–36]. *L. candidum* L., commonly known as the white lily, has been used in folk medicine and has shown potential for antimicrobial activity, which may apply to aquaculture settings [37]. *L. candidum* contains phytochemicals including kaempferol, linalool, citronellal, and humulene which have immunostimulatory potential for the secretion of pro-inflammatory cytokines IL-6 and IL-8 [37, 38]. Despite the potential of herbal extracts in controlling shrimp bacterial diseases, comprehensive studies on their efficacy in aquaculture remain limited [39, 40]. Furthermore, scientific evidence supporting the antibacterial activity of herbal extracts against pathogenic bacteria in shrimp farming is insufficient to encourage widespread adoption by aquaculturists. This study investigated the effects of three eco-friendly herbal extracts (*A. herba-alba*, *L. japonica*, and *L. candidum*) on intensively cultured *L. vannamei*. We assessed their impact on growth performance, feed utilization efficiency, and immune function parameters. Additionally, we evaluated the disease resistance of herbal-treated *L. vannamei* against *V. parahaemolyticus*.

## Materials and methods

### Ethical approval

This study adhered to animal welfare guidelines as outlined by the American Fisheries Society (AFS, 2014). The experimental procedures, including fish handling, examination, and rearing, were conducted following the recommendations and approval of the Committee for Ethical Care and Use of Animals and Aquatic Animals at the National Institute of Oceanography and Fisheries (NIOF, Egypt) under the protocol number NIOFAQ2F23R029.

### Herbal extract preparation

Fresh samples of *A. herba-alba*, *L. japonica*, and *L. candidum* (1 kg each) were obtained from the National Agriculture Research Center (NARC), Egypt. The plant materials underwent cleaning, air-drying at 40 °C for 72 h, and subsequent grinding into a powder form. For each herb, 100 g of powder was mixed with 70% ethanol (1:10 w/v) and left at ambient temperature for 72 h. The mixtures were then stirred magnetically at 150 rpm for 24 h and filtered twice using Whatman No. 1 papers (42 μm pore size). A rotary evaporator (BÜCHI Rotavapor R-124) with a water bath (B-480) was employed to remove the alcohol. The resulting concentrated extracts were stored in dark, sterile glass containers at −20 °C until further use [24, 41].

### Phytochemical analysis of crude extracts

#### Total phenolic compound determination

Total phenolics in the extracts were quantified using a UV spectrophotometer (Jenway-6405-UV/VIS) and the Folin-Ciocalteu reagent, based on a colorimetric oxidation/reduction reaction. The procedure involved mixing 0.5 mL of diluted extract with 2.5 mL of diluted Folin-Ciocalteu reagent and 2 mL of Na_2_CO_3_ solution (75 g/L) [42]. After incubation at 50 °C for 5 min and subsequent cooling, absorbance was measured at 763 nm. Distilled water was used as a control. Total phenolic content was expressed as gallic acid equivalents (GAE), calculated using the equation: y = 0.0187x + 0.0786 (R^2^ = 0.998), where (y) represents absorbance, (x) denotes concentration (mg GAE g⁻^1^ extract) and (R^2^) is the correlation coefficient (Table 1).

**Table 1:** Analysis of total flavonoids, and phenolic content of the selected plant

**Scientific names**	**Local names**	**Plant parts**	**Total flavonoids**^a^	**Total phenolic**^b^
*A. herba-alb*	*Artemisia*	leaves	2.404054	12.56898
*L. japonica*	honeysuckle	Stems, and flowers	8.431081	14.90053
*L. candidum*	*Lilium*	Bulbs, and roots	7.322973	14.28556

^a^mg QE / g dry plant: Total flavonoid contents expressed as quercetin equivalent (µg QE g^−1^ extract)

^b^mg GAE / g dry plant: Total phenolic contents expressed as gallic acid equivalent (mg GAE g^−1^ extract)

#### Determination of total flavonoids

Total flavonoid content was assessed following a modified method from Ordon et al. (2006). The process involved adding 3 mL of 10 g L⁻^1^ aluminum chloride (AlCl3) ethanolic solution to 0.5 mL of extract. After 1 h of dark incubation, absorbance was measured at 420 nm at room temperature. Extract samples were evaluated at a final concentration of 1 mg/mL. Total flavonoid content was expressed as quercetin equivalent (QE), calculated using the equation: y = 0.0148x—0.0118 (R^2^ = 0.9996), where x represents absorbance and y denotes concentration (μg QE) and R2 is the correlation coefficient (Table 1 ).

### Formulation of basal and functional diets

The basal and herbal-incorporated diets were formulated to meet the optimal nutritional requirements for *L. vannamei* (Guimaraes et al. 2008; Niu et al. 2011). All diets were designed to be isoproteic and isoenergetic. The composition included dry matter (90.39%), crude protein (40.69%), crude fat (7.36%), crude fiber (3.68%), ash (10.02%), nitrogen-free extract (NFE) (28.64%), and gross energy (18.15 MJ kg^1^) (Table 2 ). For diet preparation, dry ingredients were initially weighed, combined, and homogenized in a Hobart-type mixer. Oil was then incorporated and mixed thoroughly for 5 min. Subsequently, herbal extracts were added at a concentration of 250 mg kg^1^ and mixed with deionized hot water (40% of the dry ingredient mixture) for an additional 5 min. The resulting wet mixture was extruded through a 1.2 mm die using an electric kitchen meat grinder (Moulinex ME605131—HV8—1600 W, Paris, France). The extruded pellets were initially air-dried at room temperature (25 °C) for 3 h using an air conditioner and an electric fan in a well-ventilated laboratory. Final drying was conducted at 45 °C for 24 h. The dried diets were then stored at −20 °C until use. Four experimental diets were prepared for the study. The control diet (T1) consisted of the basal diet without any herbal supplements (Table 2 ). The *A. herba-alba*-treated diet (T2) contained the basal diet supplemented with 250 mg kg^1^ *A. herba-alba*. The *L. japonica*-treated diet (T3) comprised the basal diet with 250 mg kg^1^ *L. japonica* added. The *L. candidum*-treated diet (T4) was formulated by incorporating 250 mg kg^1^ *L. candidum* into the basal diet*.*

**Table 2:** Feed formulation and proximate chemical composition of the shrimp basal diet (% on dry matter (DM) basis)

Ingredient	gm/kg	Chemical composition	%
Soybean (45% CP)	280	Dry matter	90.39
Fish meal (65% CP)	170	Crude protein	40.69
Corn gluten	100	Digestible crude protein	36.98
Wheat (12% CP)	240	Ether extract	7.36
Rice bran (8.2% CP)	30	Crude fiber	3.68
Shrimp head meal (58% CP)	80	Ash	10.02
Meat & bone meal (48% CP)	50	NFE^**2**^	28.64
Dicalcium Phosphate	10	Calcium	2.19
Vitamin/mineral premix^**1**^	6	Available phosphorus	1.09
Fish oil	12	Gross energy (MJ/kg)^**3**^	18.15
Soybean oil	10	Digestible energy (MJ/kg)	15.59
Soy lecithin	2	Total phospholipid	2.77
Cholesterol	1	EPA (20:5n-3)	0.23
Gelatin (binder)	5	DHA (22:6n-3)	0.43
Choline chloride	1	Total n-3	0.82
Vitamin C	1	Total n-6	1.14
Salt (NaCl)	2	N3:N6	0.72
Total	1000.0		

^1^ Premix (mg kg^−1^ premix): vitamin B1 (133 mg), vitamin B2 (580 mg), vitamin B6 (410 mg), vitamin B12 (50 mg), biotin (9330 mg), vitamin A (3300 IU), vitamin D3 (410 IU), vitamin E (150 mg), choline chloride (4000 mg), vitamin C (2660 mg), inositol (330 mg), para-amino benzoic acid (9330 mg), niacin (26.60 mg), pantothenic acid (2000 mg), iron (200 mg), copper (25 mg), manganese (325 mg), iodine, cobalt (5 mg)

^2^Nitrogen free extract (NFE) = 100- (moisture % + Protein% + lipids% + Ash% + Fiber %)

^3^Gross energy, kJ/g was calculated using a value of 23.62, 39.52, and 17.15 kJ/g for protein, fat, and carbohydrate, respectively, according to Brett (1973)

### Influence of dietary herbal supplements on growth and immune responses

Shrimp post-larvae were purchased from the National Company for Fishery & Aquaculture. Before initiating the official trial, these larvae underwent a two-week acclimation period of physical, clinical, and bacteriological examinations to ensure disease-free status and to establish uniform, robust, and vigorous health conditions. This study utilized 2400 shrimp post-larvae (PL) with an initial mean weight of 0.74 ± 0.02 g. These PLs were randomly allocated to four treatment groups (T1, T2, T3, and T4), each with three replicates. Each replicate consisted of 200 PLs housed in a 2 m^3^ capacity fiberglass tank. The control group (T1) received the basal diet without supplements. The experimental groups T2, T3, and T4 were fed the basal diet supplemented with *A. herba-alba, L. japonica*, and *L. candidum*, respectively. All diets were administered at a feeding rate of 5–7% of body weight. The experiment was conducted over four months. Throughout this duration, the feeding rate was regularly adjusted based on the shrimps' increasing body weight to maintain optimal nutrition.

#### Rearing and clinical observation

Feeding was scheduled four times daily at 07:00, 11:00, 17:00, and 21:00, with a photoperiod (12L: 12D). Shrimp vitality was observed one hour after feeding with. Water exchange rates ranged from 1/3–1/2 of the tank volume every other day initially, progressing to daily exchanges two weeks before the experiment's conclusion. Tanks were equipped with aeration and filtration systems and cleaned daily by siphoning. Water parameters were measured using testing kits and electronic probes [43]. Water quality was continuously maintained including temperature at 27.7 ± 0.98°C, pH at 7.5 ± 0.42, dissolved oxygen at 6.49 ± 0.86 mg/L, and salinity at 32 ± 1.75 ppt. Shrimp were regularly inspected for the appearance of behavioral or clinical signs, including anorexia, lethargy, empty gut, expanded chromatophores, and pale hepatopancreas.

#### Growth parameters

The growth rate in weight and length of the shrimp was recorded throughout the experiment and at its conclusion. Growth performance was evaluated using several parameters. Weight gain (WG, %): WG = 100 × (final weight – initial weight) / initial weight. The specific growth rate (SGR) was determined in percent per day, computed as 100 × (ln final weight – ln initial weight) / days of feeding experiment. Feed conversion ratio (FCR): FCR = feed consumed / weight gain. Survival rate (SR) was calculated as a percentage, using the equation: 100 × (final number of shrimp / initial number of shrimp). The protein efficiency ratio (PER) was determined by dividing the shrimp weight gain by the protein intake.

### Analysis of immune parameters

#### Hemolymph Sampling

Before hemolymph collection, shrimp were subjected to a 24-h fasting period. Five shrimp specimens were randomly selected from each replicate and anesthetized using MS-222 (0.1 g L⁻^1^ tricaine methanesulfonate; Sigma-Aldrich). Hemolymph was extracted from the ventral sinus at the base of the first abdominal segment using 1-cm^3^ syringes. The collected hemolymph was divided into two portions. The first portion was placed in microtubes containing an anticoagulant solution (30 mM trisodium citrate, 0.34 M NaCl, and 10 mM EDTA; pH 7.55), with its osmolality adjusted to 780 mOsm kg⁻^1^ using glucose. This hemolymph and anticoagulant mixture was prepared at a ratio of 1:9 in a sterile tube and placed on ice [44]. The second portion was placed in a tube without anticoagulant and temporarily stored at 4°C for 12 h. It was then centrifuged at 4000 rpm for 10 min, and the resulting serum was stored at −80°C for subsequent use in biochemical and immunological assays.

#### Immune assays

Several immune parameters were assessed using the collected hemolymph samples. Total hemocyte count (THC) was determined using a hemocytometer under an inverted phase-contrast microscope [45]. Phenoloxidase (PO) activity was measured based on the formation of dopachrome from L-dihydroxyphenylalanine (L-DOPA) substrate using a spectrophotometer [46, 47]. Superoxide dismutase (SOD) activity was assessed using a modified method from [48] and further verified following the method of McCord and Fridovich [49]. Lysozyme (LYZ) activity was analyzed using the turbidimetric method as described by [46]. The phagocytic activity of hemocytes was determined in a 24-well plate and expressed as a percentage of phagocytic hemocytes to total observed hemocytes [50]. Protein concentration was measured using an ELISA microplate format (Bradford 1976). Glutathione peroxidase (GPx) activity was measured following the methodology of Flohe and Gunzler (1984). Lipid peroxidation levels were determined by measuring malondialdehyde (MDA) levels during fatty acid oxidation [51]. All spectrophotometric measurements were performed using a UV–visible spectrophotometer, and all assays were conducted in triplicate to ensure the reliability of the results.

### Evaluation of the resistance of the herbal-treated shrimp to ***V. parahaemolyticus Vp***_AHPND_

The pathogenic *V. parahaemolyticus* SHA used in this study was initially isolated from diseased *Litopenaeus vannamei* shrimp during an infection outbreak, fulfilling Koch's postulates. The diseased shrimp exhibited symptoms including anorexia, lethargy, empty gut, pale hepatopancreas, and expanded chromatophores. This *V. parahaemolyticus* SHA was identified by 16S rRNA sequencing and submitted to GenBank under accession number OP659038. The bacterial strain was purchased from the Laboratory of Marine Microbiology, National Institute of Oceanography and Fisheries (NIOF), Alexandria, Egypt.

Following the 4-month feeding period with herbal-supplemented diets, the disease resistance efficacy of treated *L. vannamei* was evaluated through a challenge against the pathogenic *V. parahaemolyticus* SHA. Before the challenge test, an LD50 assay was conducted to determine the infective dosage.

#### LD50 assay

To determine the median lethal dose (LD50), a total of 210 shrimp with an average weight of 12.54 ± 0.029 g were equally distributed into seven triplicated groups (T1, T2, T3, T4, T5, T6, and T7), with 30 shrimp per group. The *V. parahaemolyticus* SHA was cultured in tryptic soy broth (TSB) supplemented with 3% NaCl and incubated at 27°C for 24 h. The bacterial cells were then harvested by centrifugation at 10,000 rpm for 1 min at 4°C. The resulting bacterial pellet was resuspended in 0.9% NaCl solution and adjusted to a concentration of 3 × 10^8^ CFU/mL (equivalent to McFarland standard #1) to serve as the stock bacterial suspension for injection [52]. This standardized suspension was then serially diluted with PBS to obtain the required concentrations ranging from 3 × 10^3^ to 3 × 10^8^ CFU/mL.

The negative control group (T1) received an intramuscular injection of 50 µL sterile 0.9% saline solution in the third abdominal segment. The remaining groups (T2 to T7) were injected intramuscularly with 50 µL of the corresponding five serial dilutions of *V. parahaemolyticus* SHA, ranging from 3.0 × 10^3^ to 3.0 × 10^8^ CFU/mL [53]. The cumulative mortality was recorded over 72 h post-injection, and based on these results, the LD50 was determined to be 3 × 10^4^ CFU/mL.

#### Bacterial challenge

The bacterial challenge was conducted following modified procedures from [53]. A total of 150 shrimp were used in this experiment, randomly chosen from the original experimental groups, with 30 shrimp taken from each of the five groups that had received different dietary treatments during the feeding trial. The selected shrimp were then redistributed into five new triplicated groups of 10 shrimp each. These new groups were housed in 500-L capacity fiberglass tanks and designated as a negative control group (T1), a positive control group (T2), and three herbal-treated groups (T3-T5). The *V. parahaemolyticus* SHA suspension used for injection was prepared as previously described, with the concentration adjusted to 3 × 10^4^ CFU/mL based on the LD50 determination. Each shrimp in the positive control T2, and herbal-treated groups (T3, T4, and T5) received an intramuscular injection of 50 μL of this bacterial suspension in the third abdominal segment, while the negative control group received an equivalent volume of sterile 0.9% saline solution.

Throughout the one-week challenge period, water quality parameters were maintained within optimal ranges through proper aeration, filtration, regular water renewal, and adherence to hygienic practices. The experimental shrimp were fed daily with a diet containing 40% protein at a rate of 5% of their biomass. Shrimp were monitored daily for clinical signs, morbidity, and mortality. Bacteriological samples from dead and moribund shrimp were aseptically collected and cultured on thiosulfate-citrate-bile salts-sucrose (TCBS) agar to confirm *V. parahaemolyticus* as the causative agent of mortality. Mortalities were attributed to *V. parahaemolyticus* only when a pure culture of the bacterium was isolated from the challenged shrimp, thereby fulfilling Koch's postulates. This rigorous approach ensured the accurate identification of *V. parahaemolyticus* as the primary cause of observed mortalities.

#### Histopathological Examination 

Hepatopancreas samples from challenged shrimp were processed for histopathological examination following standard protocols [54, 55]. Tissues were fixed in Davidson's AFA fixative, dehydrated through a graded ethanol series, cleared in xylene, and embedded in paraffin wax. Sections of 5–6 μm thickness were prepared using a rotary microtome (KD-2258), mounted on glass slides, and stained with hematoxylin and eosin (H&E). Tissue sections were examined using a light microscope (BEL photonic), and photographs were captured using a digital camera (TOUPCAM TM-UCMOS-3.1MP1/2, Model No.3.2).

### Statistical analysis

Data were analyzed using one-way ANOVA and Duncan's multiple range test (SPSS for Windows, version 22, USA). The significance level was set at P ≤ 0.05. Standard errors were estimated. Principal Correspondence Analysis (PCA) was performed using the Brodgar statistical program (Brodgar version 2.7.5, 2017, Highland Statistics Ltd., www.highstat.com).

## Results

### Influence of dietary herbal supplements on growth performance and immune response

#### Clinical Signs and Health Indicators

The *L. vannamei* treated with herbal supplements exhibited improved health indicators compared to the control group. These shrimps displayed more active swimming patterns, enhanced appetite, and improved growth. Their body parts generally appeared clearer and more brightly colored, with a harder shell texture. Additionally, the treated shrimp showed full guts and good overall coloration without signs of melanization, all indicative of better health status.

#### Growth performance

Significant differences (P ≤ 0.05) were observed in various growth parameters among the herbal-treated and non-herbal-treated groups. The *L. candidum*-treated group demonstrated significantly higher FW, WG, SGR, RGR, and survival ratio (_*P*_ ≤ *0.05*) compared to both the *A. herba-alba*-treated and *L. japonica*-treated groups. Overall, shrimp receiving herbal supplementation showed significantly improved growth performance compared to the untreated control group, as detailed in (Table 3).

**Table 3:** Growth and survival performance of *L. vannamei* fed herbal-supplemented diet under an intensive culture system

Growth and survival Parameters^1^	Treatments^2^			
	**T1**	**T2**	**T3**	**T4**
**Initial weight (IW), g/shrimp** ^**3**^	0.743 ± 0.003	0.750 ± 0.006	0.747 ± 0.007	0.750 ± 0.006
**Final weight (FW), g/shrimp**	11.86 ± 0.003^**c**^	12.94 ± 0.029^**b**^	12.97 ± 0.075^**b**^	13.84 ± 0.052^**a**^
**Weight gain (WG), g/shrimp**	11.12 ± 0.006^**c**^	12.19 ± 0.040^**b**^	12.23 ± 0.072^**b**^	13.09 ± 0.055^**a**^
**ADG, g/shrimp/day**	0.093 ± 0.000^**c**^	0.102 ± 0.000^**b**^	0.102 ± 0.001^**b**^	0.109 ± 0.001^**a**^
**AWG, g/shrimp/week**	0.649 ± 0.0002^**c**^	0.711 ± 0.002^**b**^	0.713 ± 0.004^**b**^	0.764 ± 0.003^**a**^
**SGR, %/shrimp/day**	2.308 ± 0.004^**c**^	2.377 ± 0.008^**b**^	2.381 ± 0.003^**b**^	2.429 ± 0.008^**a**^
**RGR, %/shrimp**	1596.1 ± 9.3^**c**^	1733.6 ± 17.2^**b**^	1741.0 ± 5.7^**b**^	1845.6 ± 18.6^**a**^
**Survival, %**	62.33 ± 0.73^**c**^	74.17 ± 0.60^**b**^	75.67 ± 0.60^**b**^	83.83 ± 1.17^**a**^

^1^ *ADG* Average daily gain, *AWG* Average weekly gain, *SGR* Specific growth rate, *RGR* Relative growth rate

^2^ Treatments: T1: shrimp fed a basal diet (control) without any herbal supplements; T2: shrimp fed a basal diet supplemented with 250 mg kg^−1^ *A. herba-alba;* T3: shrimp fed a basal diet supplemented with 250 mg kg^−1^ *L. japonica;* T4: shrimp fed a basal diet supplemented with 250 mg kg^−1^ *L. candidum*

^3^ Initial weight = 0.748 ± 0.003 g/shrimp

#### Feed utilization and productivity parameters

*L. vannamei* fed herbal-treated diets exhibited significantly (_*P*_ ≤ *0.05)* enhanced feed utilization and productivity parameters compared to the non-herbal-treated group. The *L. candidum*-treated group demonstrated significantly lower feed intake, improved FCR, and a higher protein efficiency ratio than those in the *A. herba-alba* and *L. japonica*-treated groups. Consequently, the highest productivity was achieved in the *L. candidum*-treated group, followed by the *L. japonica*-treated group, and then the *A. herba-alba*-treated group (Table 4).

**Table 4:** Feed utilization and productivity parameters of *L. vannamei* fed herbal-supplemented diet under an intensive culture system

Feed utilization & Productivity parameters1	Treatments2			
	T1	T2	T3	T4
Feed Intake, gm/shrimp	23.22 ± 0.22a	21.49 ± 0.01b	20.97 ± 0.11c	18.53 ± 0.04d
FCR	2.09 ± 0.02a	1.76 ± 0.01b	1.72 ± 0.02b	1.41 ± 0.01c
PER, gm/gm	1.177 ± 0.01d	1.393 ± 0.01c	1.433 ± 0.01b	1.737 ± 0.01a
Productivity, kg/1m3	0.740 ± 0.009c	0.960 ± 0.006b	0.982 ± 0.013b	1.160 ± 0.014a

1 *FCR* Feed Conversion Ratio, *PER* Protein Efficiency Ratio

2 Treatments: T1: shrimp fed a basal diet (control) without any herbal supplements; T2: shrimp fed a basal diet supplemented with 250 mg kg−1 *A. herba-alba;* T3: shrimp fed a basal diet supplemented with 250 mg kg−*1* *L. japonica;* T4: shrimp fed a basal diet supplemented with 250 mg kg−1* L. candidum*

#### Immune response of herbal-treated *L. vannamei*

**Fig. 1:**
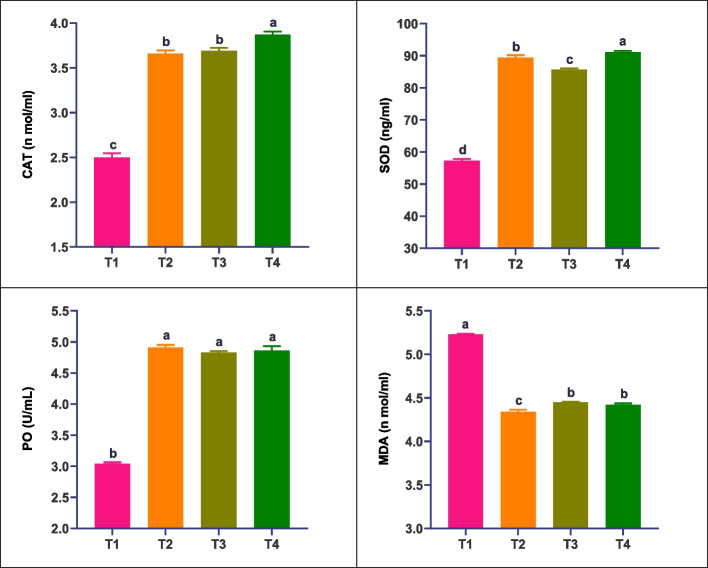
Serum antioxidative status of white leg shrimp, *L. vannamei* fed herbal-supplemented diets under an intensive culture system for 120 days. Catalase, (CAT), Superoxide Dismutase (SOD), phenol oxidase (PO), and Malondialdehyde (MDA). The bars (mean ± SE) with different letters are significantly different (*P* ≤ 0.05). Treatments: T1: shrimp fed a basal diet (control) without any herbal supplements; T2: shrimp fed a basal diet supplemented with 250 mg kg^−1^ *A. herba-alba;* T3: shrimp fed a basal diet supplemented with 250 mg kg^−1^ *L. japonica;* T4: shrimp fed a basal diet supplemented with 250 mg kg^−1^ *L. candidum*

Herbal-treated *L. vannamei* exhibited significantly enhanced (_*P*_ ≤ *0.05*) immune parameters compared to non-treated shrimp. The *A. herba-alba*, *L. japonica*, and *L. candidum*-treated groups showed increased (_*P*_ ≤ *0.05*) values of total hemocyte count, total protein concentration, and phagocytosis activity relative to the control. Glutathione peroxidase (GPx) and phenoloxidase (PO) activities were significantly higher in the *A. herba-alba*-treated group (T2) compared to other treated groups. The *L. japonica*-treated group (T3) demonstrated maximum lysozyme (LYS) activity. Higher antioxidant activity, as measured by catalase (CAT) and superoxide dismutase (SOD), was observed in the *L. candidum*-treated group (T4). Conversely, malondialdehyde (MDA) levels were significantly lower in all herbal-treated groups compared to the control group. These results, presented in Table 5 and Fig. 1, indicate that herbal extracts efficaciously enhanced the immune response of cultured whiteleg shrimp compared to the control (Fig. 1).

**Table 5:** Immune, and antioxidant parameters of *L. vannamei* fed herbal-supplemented diets under an intensive culture system

Immune parameters1	Treatments2			
	T1†	T 2‡	T 3	T 4
Total hemocyte count (106 mL − 1 hemolymph)	3.24 ± 0.029d	4.91 ± 0.035a	4.74 ± 0.046b	4.63 ± 0.058b
Phagocytosis activity (%)	17.3 ± 0.029d	39.4 ± 0.104c	42.0 ± 0.248a	40.8 ± 0.069b
Total protein (g/dL)	12.3 ± 0.06d	36.9 ± 0.29c	38.0 ± 0.46b	40.8 ± 0.05a
GPx (mg protein)	62.7 ± 0.35c	77.6 ± 0.40a	75.3 ± 0.10b	75.1 ± 0.13b
Lyz (U/mL)	0.95 ± 0.023c	1.84 ± 0.029b	1.92 ± 0.040ab	1.87 ± 0.023ab
MDA (n mol/ml)	5.23 ± 0.006a	4.34 ± 0.023d	4.45 ± 0.006b	4.42 ± 0.017bc
PO (U/L)	3.04 ± 0.02b	4.91 ± 0.04a	4.83 ± 0.02a	4.86 ± 0.07a
CAT (n mol/ml)	2.50 ± 0.05c	3.66 ± 0.03b	3.69 ± 0.03b	3.87 ± 0.04a
SOD (ng/ml)	57.3 ± 0.52d	89.4 ± 0.75b	85.7 ± 0.35c	91.1 ± 0.40a

1*GPx* glutathione peroxidase, *Lyz* Lysozyme activity, *MDA* Malondialdehyde, *GSH* Glutathione, *CAT* Catalase, *SOD* Superoxide Dismutase, phenol oxidase (PO) (U/L)

2 Treatments: T1: shrimp fed a basal diet (control) without any herbal supplements; T2: shrimp fed a basal diet supplemented with 250 mg kg*−1* *A. herba-alba;* T3: shrimp fed a basal diet supplemented with 250 mg kg−1* L. japonica;* T4: shrimp fed a basal diet supplemented with 250 mg kg−1* L. candidum*

### Bacterial challenge with *V. parahaemolyticus*

#### Mortality and survival rates

The challenge data, including cumulative mortality and relative survival percentage of *L. vannamei* challenged with *V. parahaemolyticus* (Table 6). No mortality was recorded in the negative control group (T1), which was inoculated with physiological saline, during the seven days post-challenge. Herbal supplementation significantly increased (_*P*_ ≤ *0.05*) the survival rate of challenged *L. vannamei* compared to the positive control. Lower mortality rates of 23.33%, 20%, and 13.33% were significantly (_*P*_ ≤ *0.05*) recorded in the challenged herbal-treated groups T3, T4, and T5, respectively, compared to 66.67% in the positive control (T2).

**Table 6:** Total cumulative mortality, and Relative percent survival RPS of challenged *L. vannamei* with *V. parahaemolyticus*

Parameters^1^	Treatments^2^				
	**T1**	**T2**	**T3**	**T4**	**T5**
**Initial shrimp number, #**	30	30	30	30	30
**TCM, #**	0	27.0 ± 1.0^a^	7.0 ± 0.58^b^	6.0 ± 0.58^bc^	4.0 ± 0.00^c^
**Mortality (%)**	0	66.67 ± 3.33^a^	23.33 ± 1.92^b^	20.0 ± 1.92^bc^	13.33 ± 0.00^c^
**RPS (%)**	100	-	74.074 ± 2.14	77.778 ± 2.14	85.185 ± 0.00

^**1**^ Total cumulative mortality (TCM); Relative percent survival (RPS) (Amend 1981) of challenged fish was calculated as follows: RPS = 1 – (% mortality in test group/% mortality in the control group) × 100

^**2**^ Treatments: T1: Negative-control, shrimp fed on basal diet and inoculated with physiological saline, T2: positive-control shrimp fed basal diet and challenged with *V. parahaemolyticus*, T3: shrimp fed *A. herba-alba;* supplemented diet and challenged with *V. parahaemolyticus,* T4*:* shrimp fed *L. japonica*-supplemented diet and challenged with *V. parahaemolyticus,* T5*:* shrimp fed *L. candidum*-supplemented diet and challenged with *V. parahaemolyticus*

#### Clinical signs

**Fig. 2:**
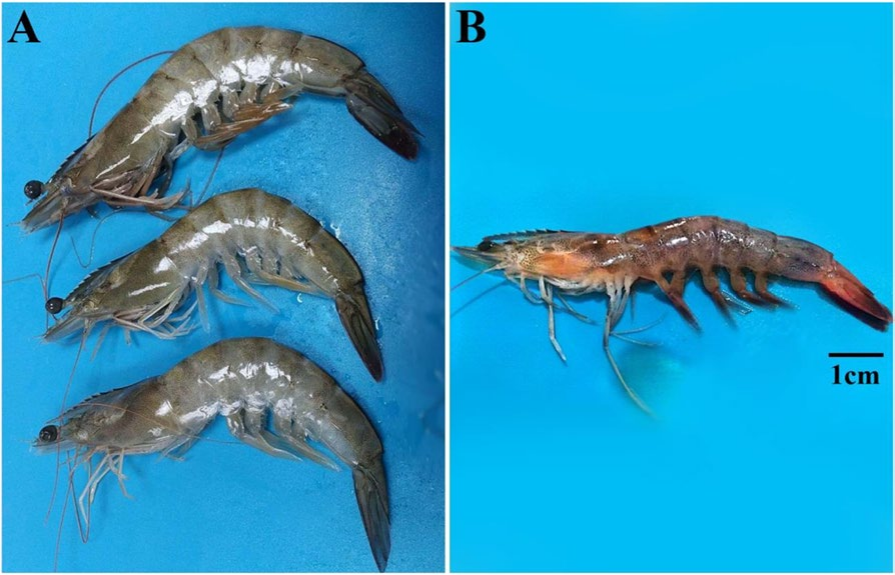
**A** Healthy *L. vannamei* with normal bright color, firm body texture, and active responsiveness before being challenged with *V. parahaemolyticus*. **B** Side view of diseased *L. vannamei* post-challenged with *V. parahaemolyticus* showing expanded chromatophores, reddened and necrotic legs, and tail fan. The scale bar = 1 cm

Post-challenge, the severity of signs associated with *V. parahaemolyticus* infection varied between herbal-treated groups and controls. In herbal-treated groups, most challenged shrimp displayed lethargy and anorexia on the second-day post-challenge. At 72 h post-challenge, some moribund shrimp exhibited expanded chromatophores, melanization, and pale hepatopancreas, with progressive mortality. In contrast, the positive control group experienced earlier signs at 12 h post-challenge, including anorexia, weakness, lethargy, and abnormal up-and-down swimming. These signs progressed to loss of feeding, weakness, immobility at the tank bottom, expanded chromatophores, melanization, and pale hepatopancreas, accompanied by continued morbidity and mortality between 24–48 h post-challenge. In all groups, morbid and dead shrimp displayed flaccid and mushy texture, discolored faded shells, whitish discoloration of the abdominal muscle, and reddened, necrotic tail fans (Fig. 2). Moribund shrimp also revealed empty guts and pale, atrophied hepatopancreas.

#### Histopathological examination

**Fig. 3:**
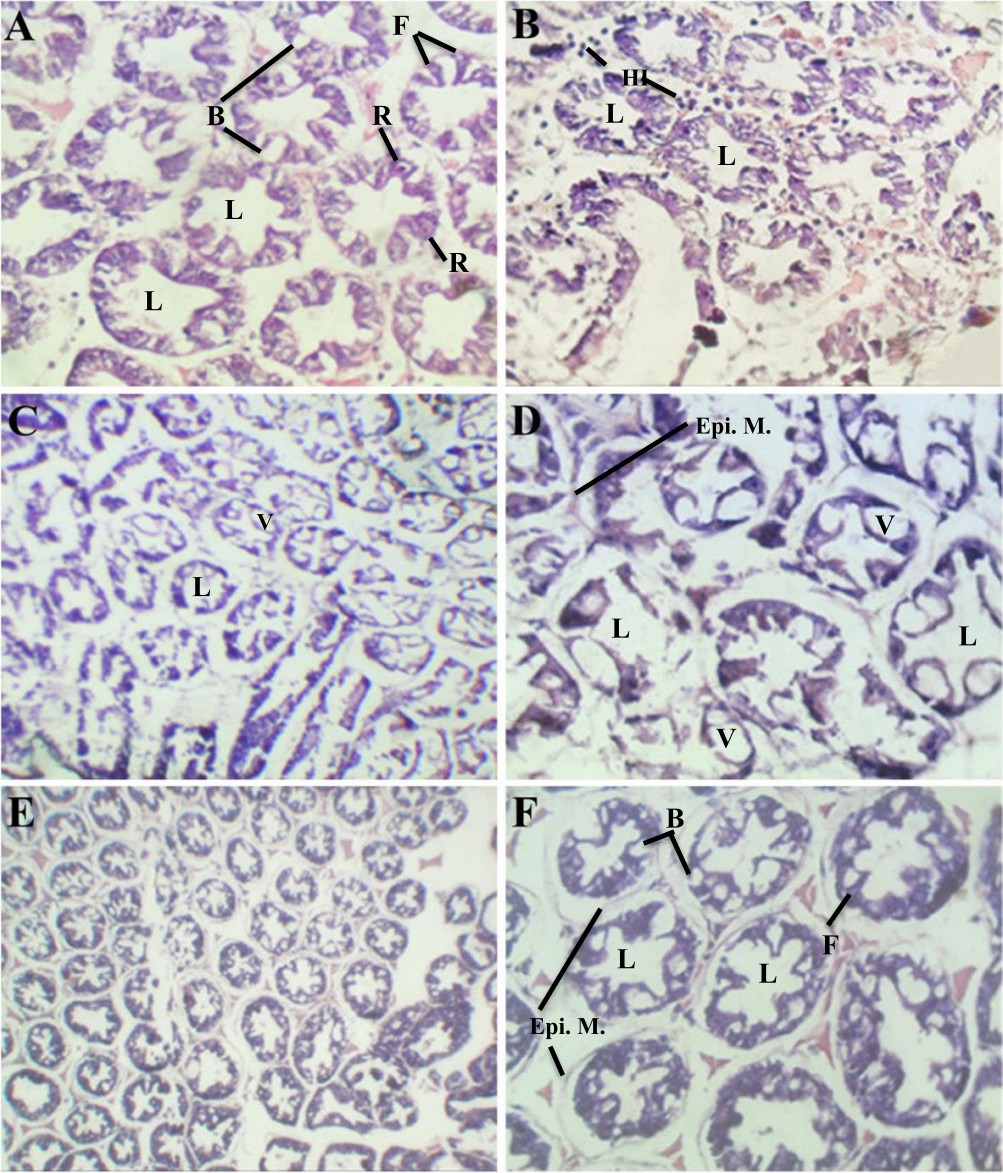
Photomicrographs of hepatopancreas in *L. vannamei.* **A** T.s in the non-challenged negative-control treatment showing normal hepatopancreatic tubules, with different cells (E, R, F& B-cells) (10 × magnification). **B** T.s in the challenged positive control treatment with degeneration in epithelial membrane and appearance of hemocytes infiltration (10 × magnification). **C**, **D** T.s in the challenged *A. herba-alba*-treated group showed mild atrophy of hepatopancreatic B-cells and R-cells with tetra-shaped star tubular lumens (10&40 × magnification). **E**, **F** T.s in the challenged *L. candidum*-treated group with mild regularly arranged hepatopancreas tissue, well developed B-cells, and F-cells detected in contact epithelial membrane around a Penta or hexa-shaped star lumenar tubules (10&40 × magnification) *Epi.M; epithelium membrane, HI; hemocytes infiltration, L; lumen, V;vacuoles*. H&E stain

Histopathological examination revealed differences in hepatopancreas tissue between challenged shrimp in non-herbal-treated and herbal-treated groups. The hepatopancreas of the negative control showed normal histological structures with firmly arranged blind tubules, narrow lumens, and intact epithelial membranes. These tubules typically exhibited a stellate structure containing four functional cell types: E-cells (embryonalzellen), R-cells (Restzellen), F-cells (Fibrillenyellen), and B-cells (Blasenzellen) (Fig. 3A). In contrast, the positive control shrimp displayed notable characteristics of *V. parahaemolyticus* infection, including atrophy, sloughing into the tubular lumen, necrosis, massive and progressive degeneration of epithelial cells in hepatopancreatic tubules, and hemocyte infiltration between the tubules (Fig. 3B).

The *A. herba-alba*-treated group showed mild atrophy and deformation of B-cells and R-cells in hepatopancreatic tubules. The lumen appeared as a tetra-shaped star occupying most of the tubule space, with irregularly arranged tubules and scattered vacuoles (Fig. 3C & D). The *L. candidum*-treated group displayed a more regularly arranged hepatopancreas tissue structure. The epithelial membrane appeared intact around the tubules, and the lumen occupied regular space, forming penta- or hexa-shaped star tubules with well-developed B-cells and F-cells (Fig. 3E & F). Notably, *V. parahaemolyticus* was not detected in the herbal-treated groups compared to the positive control. These histological changes suggest the ameliorating and recovery potential of herbal supplements in enhancing *L. vannamei* resistance against *V. parahaemolyticus* infection.

## Discussion

The cultured white-leg shrimp *L. vannamei* is highly susceptible to *Vibrio* infections which were demonstrated as potential hotspots for aquaculture sustainability and zoonotic concerns [56]. In particular, *V. parahaemolyticus* is the causative of AHPND, a destructive emerging bacterial disease affecting the shrimp aquaculture industry, causing substantial economic losses [13].

Herbal phytobiotics have emerged as a promising approach for controlling various microbial infections in fish, and shellfish [57, 58]. Herbal extracts contain high levels of bioactive substances including biomass acids, alkaloids, glycosides, polysaccharides, and flavonoids, which possess effective medicinal properties and numerous healthcare functions in animal, and human medicine [21, 59, 60]. Certain herbal extracts have already been included commercially and supplemented for aquafeeds as growth promoters, antimicrobial compounds, and sources of vital nutrients [25, 61]. However, the therapeutic effectiveness of numerous extracts in the aquaculture field has not been sufficiently investigated in terms of their usage, antimicrobial potential, immune response, growth performance, and disease resistance activities [62, 63].

Herein, the used herbal extracts; *A. herba-alba, L. japonica,* and *L. candidum* contain various active components that contribute to their immune-stimulating and antibacterial properties. *A. herba-alba* is rich in artemisinin, flavonoids, and phenolic compounds, which have been shown to possess potent antioxidant and antimicrobial activities [64]. Artemisinin, in particular, has demonstrated the ability to enhance immune function and disease resistance in aquatic species [65]. Recent studies have shown that artemisinin can effectively counteract bacterial-induced inflammation and metabolic changes in various aquatic species, highlighting its potential as a natural alternative to traditional antibiotics in aquaculture [65]. *L. japonica* contains chlorogenic acid, luteolin, and quercetin, which exhibit strong anti-inflammatory and antioxidant effects [33]. These compounds have been found to modulate immune responses and improve gut health in various animal models [66]. *L. candidum* is known for its content of steroidal saponins, alkaloids, and flavonoids such as kaempferol [37]. These compounds have shown anti-inflammatory properties and the ability to modulate immune responses [67].

Following a 4-month herbal-supplementation experiment, we observed significant improvements in growth performance, survival, feed utilization, and increased shrimp productivity in herbal-supplemented diets compared to the control, with the greatest enhancement mainly in the *Lilium*-treated diet. This evident improvement in growth performance may be partly related to the better utilization of the offered feeds in the herbal-treated groups, which is consistent with many studies [68, 69]. It has been demonstrated that herbs can improve feed palatability by stimulating the taste buds of farmed fish, resulting in increased feed consumption [60]. As well, the nucleotides, free amino acids, and alkaloids in herbal extracts are responsible for fish attractiveness [70]. Moreover, herbal products have a particular odor and flavor that play an important role in increasing feed intake, and they can accelerate the release of digestive enzymes, promote intestinal motility, and subsequently increase feed intake and feed utilization [60]. Abdel-Rahim et al. [68] have proven that total flavonoids and phenolics are the major growth promoters for *L*. *vannamei* fed sargassum aquatic plants.

Enhancement of immune response was significantly displayed in the herbal-treated *L. vannamei* compared to non-herbal-treated shrimp. Higher counts of total hemocyte, phagocytosis activity, and total protein concentration were recorded in the herbal-treated groups. In particular, the highest activities of GPx and PO were significantly observed mainly in the *Artemisia*-treated group, maximum activities of LYS in the *Lonicera*-treated group, and higher antioxidant activity (CAT and SOD) in the *Lilium*-treated group. Regardless of the herbal supplements, these significantly higher levels of SOD, CAT, GSH-Px, and PO proved a good job of the herbal extracts for removing harmful superoxide anion radicals [29, 71, 72]). In related studies on Artemisinin, the elevated antioxidant capacity could be one of the protective effects of Artemisinin achieved by superoxide anion radicals and reducing lipid peroxidation [64, 73]. Our results regarding *A. herba-alba* were in line with some recently demonstrated studies. Elevation of immune parameters was significantly recorded in *A. herba-alba*-treated *L. vannamei* [59]. The latter species fed cottonseed protein concentrate meal diets, artemisinin supplementation positively influences immune response, antioxidant capacity, growth, gut health, and disease resistance against *V. parahaemolyticus* [74]. Similarly, in common carp, *Cyprinus carpio*, *A. absinthium* was found to potentially enhance growth performance, oxidative capacity, and immune responses [32].

Given that the other two herbal extracts, *L. japonica* and *L. candidum,* have never been used yet in aquaculture, our evaluation was based on the medicinally demonstrated studies in humans and animals on the immunostimulatory, antimicrobial, and growth-promotor influences of the supplemented herbal extracts. We achieved higher levels of growth, survival, feed utilization, and shrimp productivity that could be attributed to the fact that *L. candidum* is rich in steroidal saponins, fucoidans, and alkaloids [37]. Spirostanol and furostanol saponins were isolated from the fresh bulbs of *L. candidum*. Beta-sitosterol and beta-sitosterol glucoside were isolated from the butanolic extract of petals [67]. The *Quillaja Saponaria*-supplemented diet rich in saponins, flavonoids, and phenolics boosted both growth and feed utilization in Nile tilapia [69] via enhancing digestive enzymes (amylase, lipase), as well as growth hormone. A recent study demonstrated that *L. candidum* extracts kaempferol, citronellal, and humulene have anti-inflammatory activity as they significantly decreased secretion of the pro-inflammatory cytokines interleukin IL-6 and IL-8 by senescent human pulmonary fibroblasts (HPFs) and human dermal fibroblasts (HDFs) [37].

Likewise, immunostimulatory and growth performances were achieved in the *Lonicer*a-treated *L. vannamei*. *L. japonica* was reported to have anti-inflammatory and antiviral effects that are associated with the major active components, including flavonoids and their glycosides, iridoids, triterpenoids, saponins, caffeoylquinic acids, organic acids, aromatic glycosides, lignans, volatile oils, and other compounds [75, 76]. A study in rats found that the extracts of *L. japonica* flower buds could be used as prebiotics to adjust intestinal flora with intestinal microflora imbalances [77] and 2012b). Further studies prove that the extracts of *L. japonica* flower buds could improve intestinal microbes, significantly reduce intestinal alkaline phosphatase activity, and enhance the intestinal immune barrier by regulating the content of secretory immunoglobulin SIgA and cytokines IFN-γ and IL-4 [66]. Moreover, the total flavones of *L. japonica* flower buds were proven to have hepatoprotective activity against liver damage induced by LPS in rats by reducing the production of free radicals, inhibiting cell membrane lipid peroxidation, and reducing the release of inflammatory mediators [78].

The disease resistance efficacy of herbal-treated *L. vannamei* against AHPND was proven based on the challenge potential against *V. parahaemolyticus*. Accordingly, the lowest significant mortality rate, 23.33, 20, and 13.33%, were respectively recorded in *A. herba alba, L. japonica,* and *L. candidum*-treated groups, compared to the highest mortality rate of 66.67% in the positive-control shrimp. Close challenge models of the *V. parahaemolyticus* concentration were previously demonstrated [53, 79, 80]. Post-challenge, bacteria colonize within the lymphoid, and cellular degradation can be detected about the 7th dpi (Van de Braak et al. 2002). Hence, a high bacterial load tends to overwhelm the shrimp's immune systems, and shrimp succumb to the disease (Pantoja et al. 2005). Challenged *L. vannamei* with *V. parahaemolyticus* presented clinical patterns including anorexia, lethargy, and erratic swimming, which progressed to expanded chromatophores, opaque musculature, soft shells, reddened and necrotic legs and tail, atrophied hepatopancreas, an empty gastrointestinal tract, and mortality [53],Aguilera-Rivera et al. 2019). These associated signs were a consequence of the establishment of acute hepatopancreatic necrosis disease (AHPND) in infected *L. vannamei*, *P. monodon*, *P. chinensi*, and *Marsupenaeus japonicus* [15, 81–83].

The challenged herbal-treated *L. vannamei* with *V. parahaemolyticus* presents moderate signs that disappear a few days later. As stated in the first experiment, different herbal supplements significantly improved growth performance, feed utilization, and survival rate. In addition, the enhancement of hematological and immunological indicators, including total hemocyte count, phagocytosis activity, total protein, glutathione peroxidase, lysozyme activity, and antioxidant indicators, was achieved. Subsequently, the lowest total cumulative mortality and highest relative percent survival were obtained in herbal-treated challenged *L. vannamei* with *V. parahaemolyticus* compared to the control treatment. The dose and duration of the herbal treatment are the main factors driving the enhancement in immune response and protection against pathogens. It was demonstrated that the proper dosage of dietary herbal supplements for fish and shrimp ranged from 0.1% to 10% and duration from 14 to 70 days [84].

Finally, evident improvement of histopathological indicators was revealed in the hepatopancreas of the herbal-treated challenged groups compared to the controls. These histological findings with the four functional cells resembled and were confirmed among different crustaceans [85]. The hepatopancreas is an important multifunction glandular organ in crustacea that plays a great role in food digestion, absorption, and storage in addition to the secretion of growth-related hormones in different crustacean animals [86]. Oppositely, the challenged control shrimp induced notable characteristics of *V. parahaemolyticus* infection, including atrophy, sloughing, necrosis, and massive degeneration of epithelial cells in hepatopancreatic tubule, besides lack of B, R, and F cells. Similar findings were previously reported worldwide in whiteleg *L. vannamei* affected by (AHPND) from farms in northwestern Mexico [87], in Korea [88], and Vietnam [89]. Milder histopathological changes were revealed in the herbal-treated groups compared to the positive control. Evident reduction in the severity of associated histological lesions of the hepatopancreatic tissue, including a tetra-shaped and penta- or hexa-shaped star tubular lumen of the *A. herba-alba* and *L. candidum*-treated groups, respectively. These histological changes revealed the ameliorating and recovery potential of herbal supplements to enhance the resistance of *L. vannamei* against *V. parahaemolyticus* infection.

In conclusion, our study demonstrates that dietary supplementation with *A. herba-alba, L. japonica*, and *L. candidum* effectively improves growth, survival, feed utilization, antioxidant capacity, and immune response in *L. vannamei*. The herbal additives have proven their immunoprotective and resistance-enhancing properties against AHPND-causing *V. parahaemolyticus Vp*_AHPND_. These findings highlight the potential of these herbal extracts as prophylactic and therapeutic agents against bacterial pathogens in aquaculture, offering a promising alternative to conventional antibiotics and contributing to more sustainable aquaculture practices.

## Data Availability

No datasets were generated or analysed during the current study.
